# Supplementary material for: Untargeted Metabolomics and Antioxidant Capacities of Muscadine Grape Genotypes during Berry Development
Source: Antioxidants (Basel). 2021 Jun 4;10(6):914. doi: 10.3390/antiox10060914 (PMC8230005; doi:10.3390/antiox10060914)
Supplement: Supplementary file 1 [file antioxidants-10-00914-s001.zip › antioxidants-1228156-supplementary.pdf]

Supplementary Materials

Table S1. Identified metabolites in muscadine grape genotypes at selected developmental stages.

No.	Compounds identified in ESI+ mode	Compounds identified in ESI- mode	Compounds identified in both ESI+ and ESI- mode	Fruit-Set (FS)	Veraison (V)	Ripening-Skin/Flesh (RIP-SF)	Ripening-Seeds (RIP-S)
**1**	Piperidine	Methyl acetate	Acrylic acid	Y	Y	Y	Y
**2**	Isobutyric acid	Oxalic acid	Isocrotonic acid	Y	Y	Y	Y
**3**	Alanine	Malonic acid	Phosphate	Y	Y	Y	Y
**4**	Phenol	Glyceric acid	Serine	Y	Y	Y	Y
**5**	5-Aminolevulinic acid	Phenylacetaldehyde	Pyrocatechol	Y	Y	Y	Y
**6**	Amidosulfonic acid	Oxalacetic acid	2-Furoic acid	Y	Y	Y	Y
**7**	2-Furanmethanol	Methylsuccinic acid	Fumaric acid	Y	Y	Y	Y
**8**	Cyclohexylamine	Ureidopropionic acid	Succinic acid	Y	Y	Y	Y
**9**	Tiglic acid	Deoxyribose	L-Threonine	Y	Y	Y	Y
**10**	Triethylamine	Threonic acid	Cysteine	Y	Y	Y	Y
**11**	Aminobutyric acid	Citramalic acid	4-Hydroxybenzaldehyde	Y	Y	Y	Y
**12**	Styrene	Tartaric acid	Pyroglutamic acid	Y	Y	Y	Y
**13**	Benzaldehyde	p-Hydroxyphenylacetic acid	Itaconic acid	Y	Y	Y	Y
**14**	Quinone	Gentisic acid	L-Leucine	Y	Y	Y	Y
**15**	p-Cresol	Pimelic acid	Ornithine	Y	Y	Y	Y
**16**	Histamine	7-Methylxanthine	L-Aspartic acid	Y	Y	Y	Y
**17**	Epsilon-caprolactam	5-Methoxysalicylic acid	Malic acid	Y	Y	Y	Y
**18**	Dihydrouracil	Suberic acid	2-Methylbenzoic acid	Y	Y	Y	Y
**19**	Heptanal	Ascorbic acid	Salicylic acid	Y	Y	Y	Y
**20**	Proline	Azelaic acid	4-Acetamidobutanoic acid	Y	Y	Y	Y
**21**	Levulinic acid	D-Glucuronic acid	Oxoglutaric acid	Y	Y	Y	Y
**22**	Indole	Gluconic acid	Adipic acid	Y	Y	Y	Y
**23**	Valine	Galactaric acid	Glutamine	Y	Y	Y	Y
**24**	Ethyl lactate	Jasmonic acid	L-Glutamic acid	Y	Y	Y	Y
**25**	Erythrose	Undecanedioic acid	Cinnamic acid	Y	Y	Y	Y
**26**	Acetophenone	Liqcoumarin	Mevalonic acid	Y	Y	Y	Y
**27**	Phenylethylamine	L-Cystine	L-Histidine	Y	Y	Y	Y
**28**	Niacinamide	Vanillic acid 4-O-sulfate	Oxoadipic acid	Y	Y	Y	Y
**29**	Nicotinic acid	Diflunisal	L-Phenylalanine	Y	Y	Y	Y
**30**	Guaiacol	Citrinin	Phthalic acid	Y	Y	Y	Y
**31**	Taurine	beta-D-Glucose 6-phosphate	Phosphoenolpyruvic acid	Y	Y	Y	Y
**32**	Thymine	Neuraminic acid	Gallic acid	Y	Y	Y	Y
**33**	5-Hydroxy-2-furoic acid	3-Oxohexadecanoic acid	Aconitic acid	Y	Y	Y	Y
**34**	Furaneol	16-Hydroxyhexadecanoic acid	Shikimic acid	Y	Y	Y	Y
**35**	Dihydrothymine	Stearic acid	L-Arginine	Y	Y	Y	Y
**36**	Pipecolic acid	Luteolin	Caffeic acid	Y	Y	Y	Y
**37**	2,4,6-Octatriynoic acid	Retinoic acid	Galactose	Y	Y	Y	Y
**38**	Glutaric acid	N-Undecylbenzenesulfonic acid	L-Tyrosine	Y	Y	Y	Y
**39**	Asparagine	6-Chlorocatechin	4-Pyridoxic acid	Y	Y	Y	Y
**40**	p-Cymene	Cibaric acid	N-Acetyl-L-glutamic acid	Y	Y	Y	Y
**41**	Urocanic acid	Aesculin	Citric acid	Y	Y	Y	Y
**42**	Tyrosol	Aucubin	Scopoletin	Y	Y	Y	Y
**43**	Kojic acid	Cyclodopa glucoside	Quinic acid	Y	Y	Y	Y
**44**	Indole-3-carboxaldehyde	Xanthylic acid	Ferulic acid	Y	Y	Y	Y
**45**	4-Guanidinobutanoic acid	6-Epi-7-isocucurbic acid glucoside	L-Quebrachitol	Y	Y	Y	Y
**46**	Spermidine	S-Lactoylglutathione	Syringic acid	Y	Y	Y	Y
**47**	Coumarin	Piceid	Tryptophan	Y	Y	Y	Y
**48**	Lysine	Caryoptosidic acid	12-Hydroxydodecanoic acid	Y	Y	Y	Y
**49**	L-Methionine	Sucralose	Pantothenic acid	Y	Y	Y	Y
**50**	Phenylglyoxylic acid	Uridine 5'-diphosphate	Monobutylphthalate	Y	Y	Y	Y
**51**	Carvone	Epiafzelechin 3-gallate	Sinapic acid	Y	Y	Y	Y
**52**	Guanine	Lucuminic acid	Traumatic acid	Y	Y	Y	Y
**53**	Vanillin	Epigallocatechin gallate	N-Undecanoylglycine	Y	Y	Y	Y
**54**	Citral	Isolimonic acid	Uridine	Y	Y	Y	Y
**55**	protocatechuic acid	Eujambolin	Linamarin	Y	Y	Y	Y
**56**	Arecoline	Kaempferol 3-xylosylglucoside	Deoxyinosine	Y	Y	Y	Y
**57**	Phosphoglycolic acid	Naringin	Piscidic acid	Y	Y	Y	Y

**58**	2,5-Furandicarboxylic acid	Isowertin 2''-rhamnoside	Phaseolic acid	Y	Y	Y	Y
**59**	4-Hydroxynonenal	3,5-Digalloylepicatechin	Abscisic acid	Y	Y	Y	Y
**60**	Succinylacetone	Kaempferol 7-(6''-galloylglucoside)	Naringenin	Y	Y	Y	Y
**61**	N-Acetylvaline	Epigallocatechin 3,5-digallate	Arbutin	Y	Y	Y	Y
**62**	3 Hydroxycoumarin	Hesperidin	Phloretin	Y	Y	Y	Y
**63**	Methyl cinnamate	Delphinidin 3-sophoroside	Punicic acid	Y	Y	Y	Y
**64**	Homomethionine	Rhamnazin 3-rutinoside	Linoleic acid	Y	Y	Y	Y
**65**	p-Coumaric acid	Troxerutin	Guanosine	Y	Y	Y	Y
**66**	Methionine sulfoxide	Delphinidin 3-lathyroside 5-glucoside	Catechin	Y	Y	Y	Y
**67**	Rhamnitol	Acrylic acid	Tocopheronic acid	Y	Y	Y	Y
**68**	Quinolinic acid	Pyruvic acid	Myrsinone	Y	Y	Y	Y
**69**	Pyridoxal	Hydroxypropionic acid	Caffeoylmalic acid	Y	Y	Y	Y
**70**	Isovanillic acid	2-Ketobutyric acid	Ellagic acid	Y	Y	Y	Y
**71**	Glycerol 3-phosphate	-	Quercetin	Y	Y	Y	Y
**72**	9-Oxo-nonanoic acid	-	Epigallocatechin	Y	Y	Y	Y
**73**	Acetylornithine	-	Glutathione	Y	Y	Y	Y
**74**	Cyclamic acid	-	Myricetin	Y	Y	Y	Y
**75**	Glucosamine	-	Lepidimoic acid	Y	Y	Y	Y
**76**	Benzophenone	-	Fertaric acid	Y	Y	Y	Y
**77**	Phosphohydroxypyruvic acid	-	4-Dodecylbenzenesulfonic Acid	Y	Y	Y	Y
**78**	3,4-Dihydroxymandelic acid	-	Trehalose	Y	Y	Y	Y
**79**	2-Methylaconitate	-	Chlorogenic acid	Y	Y	Y	Y
**80**	Targinine	-	Luteone	Y	Y	Y	Y
**81**	Kynurenic acid	-	Isoferulic acid 3-O-glucuronide	Y	Y	Y	Y
**82**	Diaminopimelic acid	-	Quercetin 3-arabinoside	Y	Y	Y	Y
**83**	5-Hydroxyindoleacetic acid	-	Delphinidin 3-arabinoside	Y	Y	Y	Y
**84**	Neocnidilide	-	Epigallocatechin 3-cinnamate	Y	Y	Y	Y
**85**	Tyrosine methylester	-	Epicatechin-3-gallate	Y	Y	Y	Y
**86**	Dihydroferulic acid	-	Glucobrassicin	Y	Y	Y	Y
**87**	Dopa	-	Trifolin	Y	Y	Y	Y
**88**	Capryloylglycine	-	Astilbin	Y	Y	Y	Y
**89**	Sebacic acid	-	Oleanolic acid	Y	Y	Y	Y
**90**	Daucic acid	-	Kaempferol 3-glucuronide	Y	Y	Y	Y
**91**	Panthenol	-	Quercetin 4'-glucoside	Y	Y	Y	Y
**92**	2-Methylcitric acid	-	Glycyrrhetinic acid	Y	Y	Y	Y
**93**	Jasmonic acid	-	Neoglucobrassicin	Y	Y	Y	Y
**94**	Dihydrojasmonic acid	-	Myricetin 3-galactoside	Y	Y	Y	Y
**95**	Valylproline	-	Epigallocatechin 3'-glucuronide	Y	Y	Y	Y
**96**	3-Oxododecanoic acid	-	Asiatic acid	Y	Y	Y	Y
**97**	Tridecanoic acid	-	Medicagenic acid	Y	Y	Y	Y
**98**	N-Acetylserotonin	-	Protohypericin	Y	Y	Y	Y
**99**	Dihydrosinapic acid	-	Dicaffeoylquinic acid	Y	Y	Y	Y
**100**	Resveratrol	-	Isolariciresinol 4'-O-beta-D-glucoside	Y	Y	Y	Y
**101**	2-Isopropyl citrate	-	Quercetin 3-(6''-malonyl-glucoside)	Y	Y	Y	Y
**102**	Pirbuterol	-	Isorhamnetin 3-(6''-malonylglucoside)	Y	Y	Y	Y
**103**	Muramic acid	-	Procyanidin B1	Y	Y	Y	Y
**104**	Palmitic amide	-	Rutin	Y	Y	Y	Y
**105**	Glycerophosphocholine	-	Isochesnatin	Y	Y	Y	Y
**106**	Coumaperine	-	Limocitrin 3-rutinoside	Y	Y	Y	Y
**107**	Glucose 1-phosphate	-	Procyanidin C1	Y	Y	Y	Y
**108**	gamma-Glutamylleucine	-	-	Y	Y	Y	Y
**109**	Epidermin	-	-	Y	Y	Y	Y
**110**	Adenosine	-	-	Y	Y	Y	Y
**111**	L-Agaritine	-	-	Y	Y	Y	Y
**112**	Octadecylamine	-	-	Y	Y	Y	Y
**113**	Fukiic acid	-	-	Y	Y	Y	Y
**114**	Stearidonic acid	-	-	Y	Y	Y	Y
**115**	Linoleamide	-	-	Y	Y	Y	Y
**116**	Oleamide	-	-	Y	Y	Y	Y
**117**	Octadecanamide	-	-	Y	Y	Y	Y
**118**	Kaempferol	-	-	Y	Y	Y	Y

**119**	Argininosuccinic acid	-	-	Y	Y	Y	Y
**120**	Dihydrotestosterone	-	-	Y	Y	Y	Y
**121**	Pantoyllactone glucoside	-	-	Y	Y	Y	Y
**122**	Palmitoleoylethanolamde	-	-	Y	Y	Y	Y
**123**	Sphingosine	-	-	Y	Y	Y	Y
**124**	Sphinganine	-	-	Y	Y	Y	Y
**125**	Eicosapentaenoic acid	-	-	Y	Y	Y	Y
**126**	2'-Deoxymugineic acid	-	-	Y	Y	Y	Y
**127**	Eicosadienoic acid	-	-	Y	Y	Y	Y
**128**	N-Acetylneuraminic acid	-	-	Y	Y	Y	Y
**129**	Ethyl oleate	-	-	Y	Y	Y	Y
**130**	Caftaric acid	-	-	Y	Y	Y	Y
**131**	Isorhamnetin	-	-	Y	Y	Y	Y
**132**	Phytosphingosine	-	-	Y	Y	Y	Y
**133**	Linoleoyl ethanolamide	-	-	Y	Y	Y	Y
**134**	N-Glycolylneuraminic acid	-	-	Y	Y	Y	Y
**135**	Oleoylethanolamide	-	-	Y	Y	Y	Y
**136**	Dihydromelilotoside	-	-	Y	Y	Y	Y
**137**	Dopamine glucuronide	-	-	Y	Y	Y	Y
**138**	Glucogallin	-	-	Y	Y	Y	Y
**139**	Prostaglandin B1	-	-	Y	Y	Y	Y
**140**	Olopatadine	-	-	Y	Y	Y	Y
**141**	3-O-p-Coumaroylquinic acid	-	-	Y	Y	Y	Y
**142**	Erucic acid	-	-	Y	Y	Y	Y
**143**	Rutagravine	-	-	Y	Y	Y	Y
**144**	Lactosamine	-	-	Y	Y	Y	Y
**145**	Pelargonidin 3-rhamno-side	-	-	Y	Y	Y	Y
**146**	Trehalose 6-phosphate	-	-	Y	Y	Y	Y
**147**	Theaflavic acid	-	-	Y	Y	Y	Y
**148**	Folinic acid	-	-	Y	Y	Y	Y
**149**	Pubescenol	-	-	Y	Y	Y	Y
**150**	Proanthocyanidin A2	-	-	Y	Y	Y	Y
**151**	8,8'-Methylenebiscatechin	-	-	Y	Y	Y	Y
**152**	3-trans-p-Coumaroylrotundic acid-		-	Y	Y	Y	Y

Y: Identified, FS: fruit-set stage, V: veraison stage, RIP-SF: ripening skin/flesh stage, and RIP-S: ripening seeds stage.
